# Acknowledgment to Reviewers of *Dentistry Journal* in 2021

**DOI:** 10.3390/dj10020018

**Published:** 2022-01-27

**Authors:** Dentistry Journal Editorial Office

**Affiliations:** MDPI AG, St. Alban-Anlage 66, 4052 Basel, Switzerland

Rigorous peer-reviews are the basis of high-quality academic publishing. Thanks to the great efforts of our reviewers, *Dentistry Journal* was able to maintain its standards for the high quality of its published papers. Thanks to the contribution of our reviewers, in 2021, the median time to first decision was 19 days and the median time to publication was 44 days. The editors would like to extend their gratitude and recognition to the following reviewers for their precious time and dedication, regardless of whether the papers they reviewed were finally published:
Adrian CurtoLoredana Liliana HurjuiAkimasa TsujimotoLorel BurnsAlberto Di BlasioLuca EspositoAlberto Rodríguez-ArchillaLuís Giner-TarridaAldo Bruno GiannìLuís ProençaAlessandra LaforgiaLyudmila Bel’skayaAlessandro AntonelliMaciej MilanowskiAlessandro NotaMafalda LaranjoAna CoelhoMagda Trinajstić ZrinskiAna IvaniševićMaher AlmasriAna VukovicMarco MiglioratiAnahid Ahmadi BirjandiMarco NisiAndrea ButeraMaria Aparecida Neves JardiniAndrew BaruthMaría Arregui GambúsAnida Maria BabtanMaría De Las Nieves GonzálezAnqi ZhangMaria Del Carmen Llena PuyAntonija TadinMaria Giulia NosottiAnurag SatpathyMaria Helena FigueiralArturo Sanchez-PerezMaría MeloAsim Al-AnsariMarina Consuelo VitaleAya MitaniMárk FráterBartosz DalewskiMark HectorBeatriz Pardal-PeláezMarko MatijevićBhaven ModhaMartin KapitánBlanca Ríos-CarrascoMassimo CarossaBruno ChrcanovicMaurizio BossuCarlo GalliMeghashyam BhatCarlos Alberto Antunes ViegasMehmet GülCarlos CoboMelanie NasseripourCarlos Garaicoa-PazminoMelinda SzekelyCarlos Miguel MartoMichael KnitschkeCatherine BissonMichela RelucentiChih Yuan FangMiguel CardosoChristopher V. HughesMihai CenariuCiavoi GabrielaMing-Lun HsuClaire Egloff-JurasMircea RivișConstanze OlmsMohammed BindakhilCorina Marilena CristacheMonica MoneaCristian ScheauMuhammad ZafarCristina Meniz GarciaMutahira LoneCristina SavencuNaiara OzamizCsongor KissNarayan GandedkarDae-Woo LeeNataša Ivančić JokićDaniel Demétrio Faustino-SilvaNeamat Hassan AbubakrDanko BakarčićNicola BaldiniDario Di NardoNidal JaradatDebora FranceschiNigel D. RobbDhelal Al-RudainyNikolaos A. ChrysanthakopoulosDivesh SardanaNir UzielDobó Nagy CsabaOk Hyung NamDomagoj GlavinaPantelis KourosDomenico DalessandriPao-Hsin LiuDorin GheorghePaola GandiniElismauro Francisco MendonçaPaulo J. PalmaEsma DogramaciPaulo Jorge PalmaFabiana SokiPetar OzretićFarhin KatgePetros MourouzisFausto ZampariniPhilip W. WertzFernando NogueiraPierre-Yves Collart-DutilleulFrancesca ZaraPing LiFrancesco BennardoPiotr WychowańskiFrancois LefebvreRabia Sannam KhanGaetano IsolaRaja VeerapandianGennaro RuggieroRajesh BhardwajGiacomo OteriRamanarayanan VenkitachalamGianmaria D’AddazioRasa MladenovicGiulia BrunelloRiccardo MonterubbianesiGiuseppe Lo GiudiceRızacan SarıkayaGiuseppina NoccaRoberta GasparroGrzegorz ChladekRod MooreGrzegorz TrybekRosario SerpicoGuillermo Pradíes RamiroRui FalachoGustavo Fabián MolinaRussell KabirGustavo FernandesSalvatore BocchieriGyörgy SzabóSalvatore ScaccoHaidar HassanSamir ČimićHervé TasserySara BernardiHimanshu AroraSaturnino Marco LupiHiroyasu KoizumiSebastian BürkleinHyeonjong LeeSekyung OhIcíar ArteagoitiaSerena BianchiIlaria CampioniSeung-Il ShinImran FarooqShariel SayardoustIoana DemetrescuSheng-Wei FengIonela Ruxandra SfeatcuShintaro SukegawaIvan Onone GialainSilvia PizziJae-Hong KimSimone GalloJean-Marc RetrouveySimonetta D’ErcoleJesús PeláezSomasundaram PrasadhJie LiuSompop BencharitJiiang-Huei JengSpyros PapageorgiouJoão M. SantosStefano PaganoJoão Manuel Mendes CaramesStefano SivolellaJohn Patrik BurkhardSusan J. JenkinsJorge N.R. MartinsTakuichi SatoJulia WillettTarek El-BialyJustyna Opydo-SzymaczekTerence A. ImberyKabilan VelliyagounderThais PereiraKacper NijakowskiTing-Hsun LanKarolina Elzbieta Kaczor-UrnanowiczTomasz M. KarpińskiKassapa EllepolaVaishnavi RatheeshKatarzyna Mocny-PachońskaVerônica Pereira De LimaKe WangVictor-Vlad CostanKelvin Ian AfrashtehfarVinayak JoshiKeyvan MoharamzadehWei-Chun LinKhaled KhalafWolf-Dieter GrimmKivovics MártonYin-Hwa ShihKlara SaczukYoshikazu KobayashiKristina PerošYuri Leonidovich VasilievKunito OkuyamaYves ReingewirtzLaura Cristina RusuZahi BadranLebon NicolasZoran KarlovićLeonardo ManciniZornitsa MihaylovaLeonzio FortunatoZvonimir Uzarevic


